# Investigation of *Daphnia magna* Sub-Lethal Exposure to Organophosphate Esters in the Presence of Dissolved Organic Matter Using ^1^H NMR-Based Metabolomics

**DOI:** 10.3390/metabo8020034

**Published:** 2018-05-19

**Authors:** Vera Kovacevic, André J. Simpson, Myrna J. Simpson

**Affiliations:** 1 Department of Chemistry, University of Toronto, 80 St. George Street, Toronto, ON M5S 3H6, Canada; vera.kovacevic@mail.utoronto.ca (V.K.); andre.simpson@utoronto.ca (A.J.S.); 2 Environmental NMR Centre and Department of Physical and Environmental Sciences, University of Toronto Scarborough, 1265 Military Trail, Toronto, ON M1C 1A4, Canada

**Keywords:** tris(2-chloroethyl) phosphate, tris(2-butoxyethyl) phosphate, triphenyl phosphate, organic contaminants, water flea

## Abstract

Organophosphate esters (OPEs) are frequently detected in aquatic environments. Hydrophobic OPEs with high octanol-water partition coefficients (Log K_OW_) will likely sorb to dissolved organic matter (DOM) and consequently alter OPE bioavailability and sub-lethal toxicity. ^1^H nuclear magnetic resonance (NMR)-based metabolomics was used to evaluate how DOM (5 mg organic carbon/L) alters the metabolic response of *Daphnia magna* exposed to sub-lethal concentrations of three individual OPEs with varying hydrophobicity. *D. magna* exposed to the hydrophilic contaminant (Log K_OW_ = 1.43) tris(2-chloroethyl) phosphate (TCEP) did not have substantial metabolic changes and DOM did not alter the metabolic response. There were significant increases in amino acids and a decrease in glucose from exposure to the hydrophobic contaminant (Log K_OW_ = 3.65) tris(2-butoxyethyl) phosphate (TBOEP) which DOM did not mitigate, likely due to the high sub-lethal toxicity of TBOEP. Exposure to DOM and the hydrophobic contaminant (Log K_OW_ = 4.76) triphenyl phosphate (TPhP) resulted in a unique metabolic response which was unlike TPhP only exposure, perhaps because DOM may be an additional stressor with TPhP exposure. Therefore, Log K_OW_ values may not always predict how sub-lethal contaminant toxicity will change with DOM and there should be more consideration to incorporate DOM in sub-lethal ecotoxicology testing.

## 1. Introduction

Dissolved organic matter (DOM) is ubiquitous in natural waters and has a complex molecular structure that originates from biomass-derived precursors such as cellulose, tannin, lipids, lignin, and proteins [1]. Dissolved organic carbon (DOC) concentration is typically in the range of 1–10 mg DOC/L and can often reach 30 mg DOC/L in freshwater lakes [2]. DOM also attenuates light density along the water column which is needed for primary production and is a food source for several heterotrophic microorganisms [3]. An unfavourable impact of DOM is that it may cause stress-induced responses in freshwater organisms such as increased oxidative damage and reduction in the amount of available energy [4] as well as reduction in antioxidant capacity [5]. DOM can also bind with organic contaminants and this may alter the bioavailability and toxicity of these contaminants [6,7,8].

As the DOC concentration increases, the fraction of a contaminant bound to DOM increases and the freely dissolved fraction of a contaminant is lowered [8]. Since the freely dissolved fraction of a contaminant is bioavailable, more contaminant sorption to DOM usually decreases contaminant bioavailability and toxicity [7,8]. Much of the research regarding how DOM alters the toxicity of organic contaminants has focused on hydrophobic organic contaminants due to their strong sorption to DOM through hydrophobic partitioning [9]. Higher DOC-water partitioning coefficients (Log K_DOC_) are typically found for hydrophobic contaminants that have high octanol-water partition coefficients (Log K_OW_) [9,10]. The sorption of hydrophobic organic contaminants to DOM has been reported to lower bioavailability and toxicity [6,7,8]. For instance, the bioaccumulation of benzo[k]fluoranthene and the activity of the biomarker for chemical exposure 7-ethoxysorufin-o-deethylase (EROD) in medaka decreased with an increase in DOM concentration [6]. In contrast, DOM was found to increase the bioavailability of organic contaminants such as pyrene and perfluoroalkyl substances to freshwater crustaceans [11,12]. Therefore, DOM may not always reduce the toxicity of organic contaminants and molecular-level mechanisms should be investigated to obtain a better understanding of how DOM may alter the toxicity of organic contaminants. 

Organophosphate esters (OPEs) are contaminants of emerging concern because they are routinely detected in freshwater ecosystems such as the Great Lakes [13]. The concentrations of some commonly detected OPEs in the Great Lakes are in the range of 0.28–1.5 ng/L of tris(2-chloroethyl) phosphate (TCEP), 2.6–75 ng/L of tris(2-butoxyethyl) phosphate (TBOEP), and 0.13–1.2 ng/L of triphenyl phosphate (TPhP) [13] and OPE concentrations in surface waters can reach up to 250 ng/L [14]. A major source of OPE contamination is from wastewater treatment plants where approximately 50% of OPEs are not removed and enter freshwater ecosystems via wastewater effluents [15]. Most ecotoxicological studies of chronic exposure of OPEs to the freshwater model organism *Daphnia magna* have reported detrimental changes in observable endpoints such as survival, reproduction, and growth [16,17,18]. The molecular-level toxicity of OPEs to *Daphnia magna* has also been evaluated by several omics methods which investigate mechanisms of sub-lethal toxicity [16,18,19,20]. This included transcriptomics of TBOEP exposure [16], tris(1,3-dichloro-2-propyl) phosphate (TDCIPP) exposure [19], and TPhP exposure [18], as well as transcriptomics, metabolomics and lipidomics of TPhP exposure as a mixture with three other contaminants [20]. For example, chronic 21-day exposure of *D. magna* to sub-lethal concentrations (14.7–1470 μg/L) of TBOEP significantly altered genes involved in protein metabolism, biosynthesis, and energy metabolism, suggesting that TBOEP alters these fundamental cellular systems [16]. In another study, *D. magna* exposed to 215 and 3534 ng/L of TDCIPP had significantly down-regulated genes involved in growth after 6 days of exposure, significantly altered genes involved in reproduction after 32 days of exposure, and significantly upregulated genes involved in cardiomyopathy after 62 days of exposure [19]. Thus, omics methods are being used to better understand the mechanism of action and evaluate the toxicity of OPE exposure at sub-lethal levels.

The influence of DOM on the toxicity of OPEs has been studied using traditional toxicity endpoints such as mortality [8]. In contrast, how DOM alters more sensitive molecular-level toxicity parameters such as transcriptional, protein or metabolic responses to organic contaminant exposure has not been studied in detail [6]. In this study, proton nuclear magnetic resonance (^1^H NMR)-based environmental metabolomics was used to evaluate how the presence of DOM changes the metabolic responses of *D. magna* exposed to three individual OPEs with varying hydrophobicity. A metabolomics approach was taken because analysing the changes of low molecular weight metabolites in cells, tissues or whole organisms provides information on the biochemical perturbations caused by contaminants and can reveal the mechanism of action of contaminants [21,22]. ^1^H NMR-based metabolomics is reproducible, gives highly quantitative assessments and a variety of metabolites can be measured with straight-forward sample preparation [23,24]. ^1^H NMR-based metabolomics has been successfully used to differentiate metabolic profiles and to discover metabolite biomarkers of aquatic invertebrates exposed to contaminants, for instance in analysing the metabolome of bivalve molluscs exposed to areas of petrochemical contamination [25,26]. In this study, *D. magna* were exposed for 48 h to one concentration of TCEP, TBOEP or TPhP in the absence and presence of DOM (5 mg DOC/L). *D. magna* was used as the test organism because this freshwater crustacean is a keystone species in aquatic ecosystems with a short life cycle and parthenogenetic reproduction and is sensitive to environmental contaminants [27]. We chose these three OPEs because they have diverse chemical structures and Log K_OW_ values that are expected to impact sorption to DOM as well as the toxicity to *Daphnia* (Table 1). We hypothesized that DOM would lessen or prevent the metabolic disturbances caused by exposure to the hydrophobic OPEs TBOEP (Log K_OW_ = 3.65) and TPhP (Log K_OW_ = 4.76) because they may become less bioavailable due to their strong sorption to DOM, as seen by their high predicted K_DOC_ values (Table 1). We also predicted that DOM would not greatly attenuate the metabolic response to exposure of the hydrophilic contaminant TCEP (Log K_OW_ = 1.43) because TCEP likely has weak sorption to DOM, as seen from its low predicted K_DOC_ value (Table 1). The objective of this study was to evaluate how DOM alters the molecular-level toxicity of OPEs with different hydrophobicity.

## 2. Results and Discussion

### 2.1. Metabolic Response in the Presence of DOM

DOM can have both beneficial and unfavourable impacts on the physiology of aquatic invertebrates [4,31]. For example, the inclusion of a low concentration of 5 mg/L Suwannee River natural organic matter with cypermethrin exposure decreased the activities of transformation enzyme systems of *D. magna* compared to cypermethrin only exposure, indicating a mitigating capacity of DOM [31]. However, a recent study has shown that increases in DOC concentration hindered reproduction, increased oxidative stress, and reduced energy stores in *D. magna* [4]. Therefore, to consider the possible impacts of DOM alone on the metabolome of *D. magna*, we compared the metabolic responses of the 5 mg DOC/L control to the control group (e.g., no DOM and no OPEs). The Principal Component Analysis (PCA) scores plot showed the 5 mg DOC/L control had no statistically significant separation from the control group (Supplementary Materials Figure S1A). This suggests that DOM did not have a significant impact on the overall metabolic profile of *D. magna* under the experimental settings of this study. Individual metabolite comparisons revealed that with DOM, a significant (*p* < 0.05) increase in alanine and a decrease in serine was observed (Supplementary Materials Figure S2). A previous metabolomics study showed that chronic exposure to 4 mg DOC/L resulted in increased amino acid levels in *Daphnia pulex-pulicaria* and it was postulated that this could be from minor oxidative stress or increased energy reserves in the presence of DOM [32]. However, our work was an acute exposure to DOM isolated from a different location, and there can be differences in sensitivity to DOM between different *Daphnia* species [4]. In our study, DOM did not significantly disrupt the overall metabolome of *D. magna*, except for slight changes in alanine and serine.

### 2.2. Metabolic Response to OPE Exposures in the Absence of DOM

**Figure 1:**
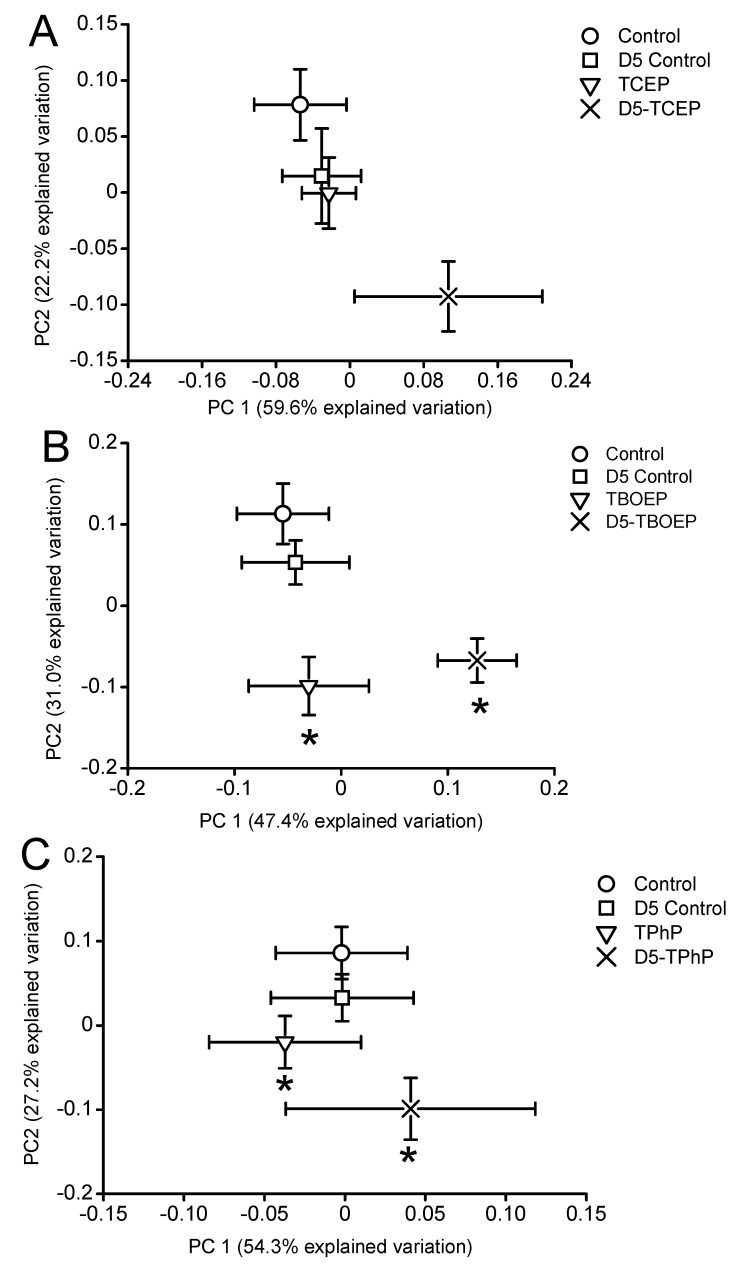
Principal component analysis (PCA) scores plots of the metabolic profiles of *Daphnia magna* exposed to (**A**) TCEP, (**B**) TBOEP, or (**C**) TPhP in the absence and presence of DOM. Average PCA scores are shown with their associated standard error. DOM control groups at 5 mg DOC/L are marked as “D5 Control”. Contaminants equilibrated with 5 mg DOC/L are marked as “D5-contaminant”. * *p* < 0.05 is between control and OPE exposed groups or between DOM control and OPE with DOM exposed groups.

**Figure 2:**
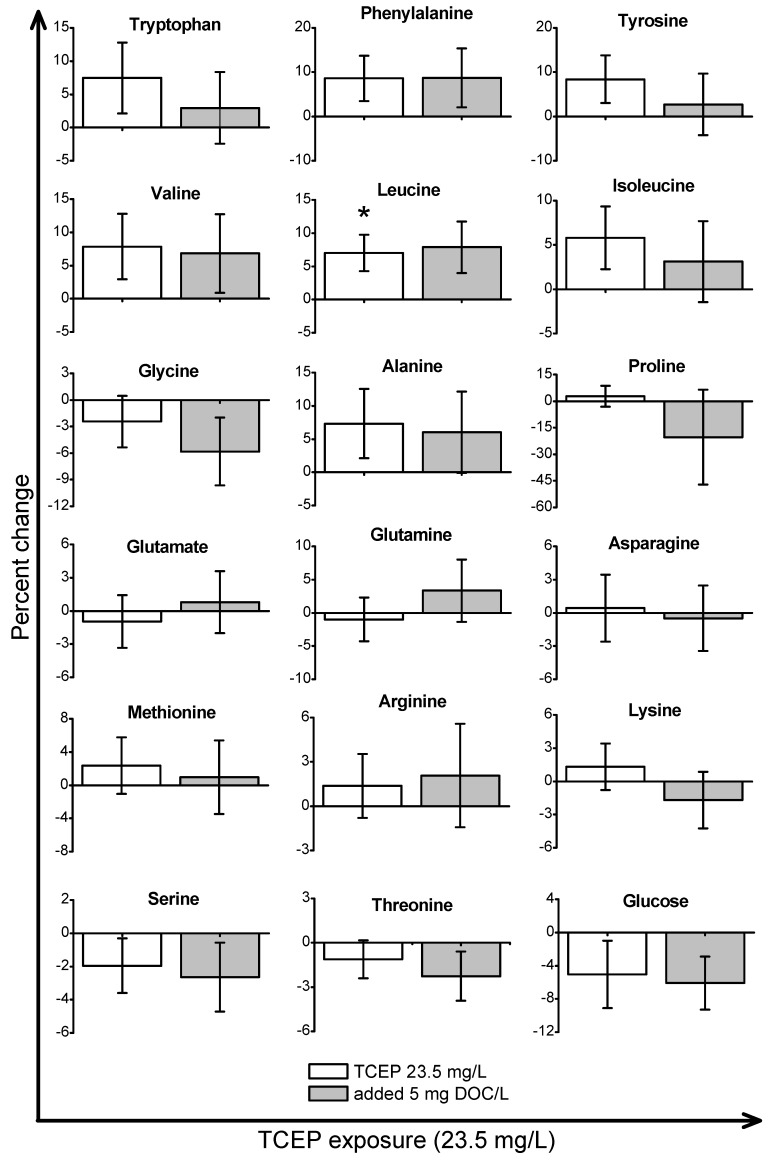
The metabolite percent changes of *Daphnia magna* exposed to TCEP in the absence and presence of DOM. The x-axis is the TCEP exposure concentration and the y-axis is the percent change of the metabolites. The TCEP only percent changes are relative to the control and the TCEP with 5 mg DOC/L percent changes are relative to the 5 mg DOC/L control. Values are shown as mean ± standard error and * represents *p* < 0.05.

**Figure 3:**
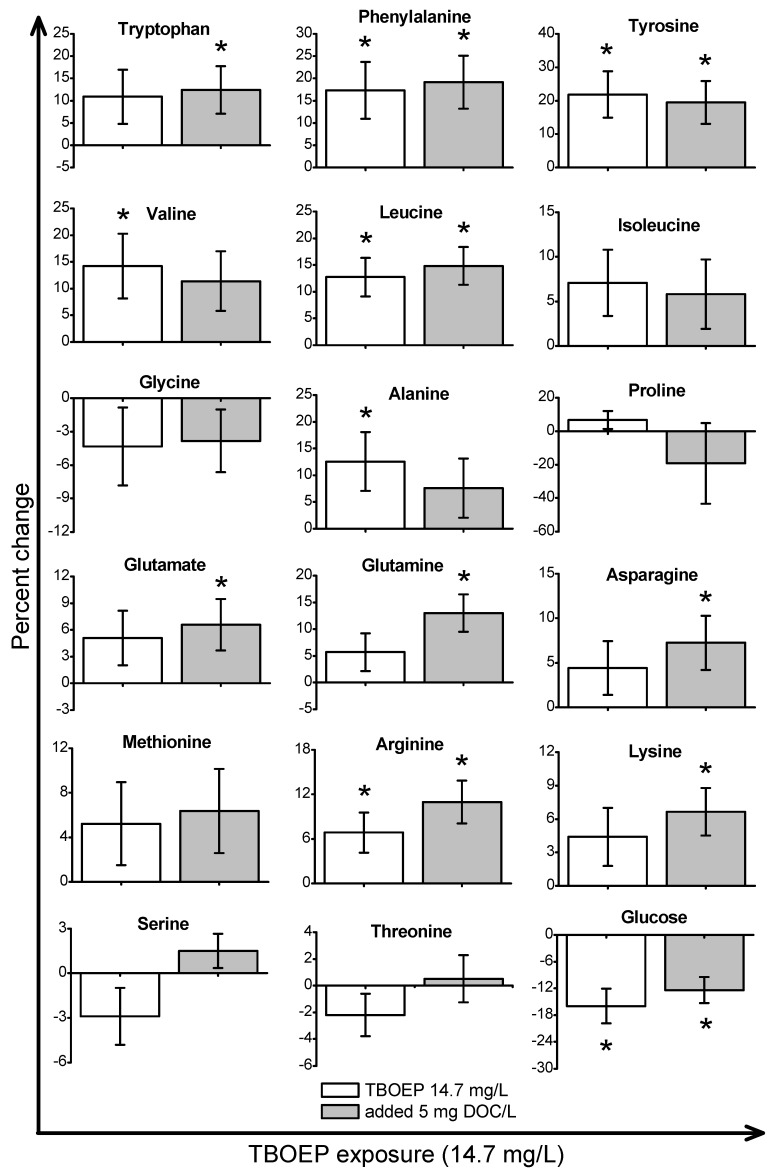
Metabolite percent changes of *Daphnia magna* exposed to TBOEP in the absence and presence of DOM. The x-axis is the TBOEP exposure concentration and the y-axis is the percent change of the metabolites. The TBOEP only percent changes are relative to the control and the TBOEP with 5 mg DOC/L percent changes are relative to the 5 mg DOC/L control. Values are shown as mean ± standard error and * represents *p* < 0.05.

**Figure 4:**
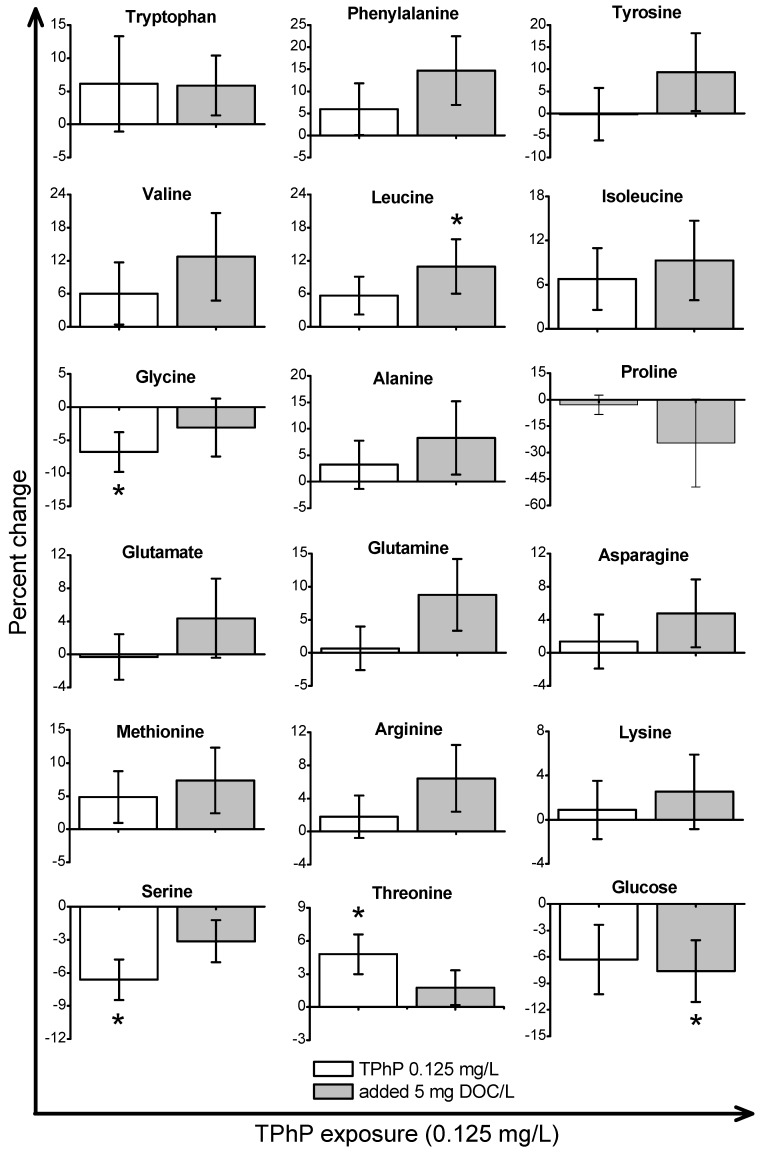
The metabolite percent changes of *Daphnia magna* exposed to TPhP in the absence and presence of DOM. The x-axis is the TPhP exposure concentration and the y-axis is the percent change of the metabolites. The TPhP only percent changes are relative to the control and the TPhP with 5 mg DOC/L percent changes are relative to the 5 mg DOC/L control. Values are shown as mean ± standard error and * represents *p* < 0.05.

*Daphnia magna* were exposed to sub-lethal concentrations of TCEP (23.5 mg/L), TBOEP (14.7 mg/L) and TPhP (0.125 mg/L). The sub-lethal exposure concentrations of TBOEP and TPhP are 10% of the 48-h lethal concentration to 50% of the population (LC_50_) values for *D. magna* (Table 1) [8,30]. The sub-lethal exposure concentration of TCEP is 10% of the reported 24-h half maximal effective concentration (EC_50_) of TCEP to *D. magna* as the 48-h LC_50_ of TCEP to *D. magna* is not reported in the literature (Table 1) [29]. PCA scores plots were used to evaluate the variability in the *D. magna* metabolome after exposure to TCEP, TBOEP and TPhP (Figure 1A–C). The TCEP exposure group did not separate from the control (Figure 1A), suggesting that TCEP exposure did not substantially change the metabolome. There was only a significant (*p* < 0.05) increase in leucine with 23.5 mg/L TCEP exposure (Figure 2), which suggests that this concentration of TCEP did not interfere with the metabolism of *D. magna*. TCEP did not greatly alter the metabolome likely because the potency of TCEP to *D. magna* is low at the tested concentration of 23.5 mg/L. The TBOEP exposure group was significantly (*p* < 0.05) separated from the control in the PCA scores plot (Figure 1B), suggesting that TBOEP exposure altered the metabolome of *D. magna*. The separation of the TBOEP exposure group from the control in the individual PCA scores plot (Supplementary Materials Figure S4A) was due to the metabolites alanine and leucine that were separated on the corresponding PCA loadings plot (Supplementary Materials Figure S4B). There were significant (*p* < 0.05) increases in six amino acids and a significant (*p* < 0.05) decrease in glucose with TBOEP exposure (Figure 3). The decrease in glucose suggests that there was a depletion of immediate energy reserves in the form of carbohydrates and increases in amino acids has often been accredited to the degradation of proteins for energy [33]. Likewise, a previous study with *D. magna* has shown that chronic 21-day exposure to sub-lethal concentrations (0.0147–1.47 mg/L) of TBOEP alter gene transcription related to energy metabolism and protein metabolism [16]. The TPhP exposure group was also significantly (*p* < 0.05) separated from the control in the PCA scores plot (Figure 1C), indicating that TPhP exposure disrupted the *D. magna* metabolome. TPhP exposure resulted in significant (*p* < 0.05) decreases in serine and glycine and a significant (*p* < 0.05) increase in threonine (Figure 4). Glycine, serine, and threonine metabolism is important for many essential cellular pathways involved in energy generation, such as gluconeogenesis [34]. In crustaceans, the decrease in glycine and serine levels could suggest that glycine is being readily converted to serine, and serine is being converted to pyruvate for use as an energy source in the Krebs cycle [35]. TPhP likely disturbed the *D. magna* metabolome as TPhP is a highly toxic and potent OPE to *D. magna* [36]. Chronic 21-day exposure of *D. magna* to 0.5 mg/L TPhP resulted in significantly decreased survival rates and body lengths, reduced production of neonates as well as significantly altered genes involved in cellular processes and metabolism [18]. The metabolic changes in *D. magna* after acute TPhP exposure may represent the onset the detrimental changes that occur in chronic 21-day TPhP exposure [18].

### 2.3. Metabolic Response to OPE Exposures in the Presence of DOM

The goal of this work was to evaluate how DOM impacts the molecular-level toxicity of three OPEs with varying hydrophobicities. TCEP is a hydrophilic compound with a relatively high water solubility and a low K_OW_ (Table 1), and hence TCEP should not have a strong capacity for DOM sorption as compared to high Log K_OW_ OPEs [8]. The TCEP with 5 mg DOC/L exposure group did not significantly separate from the 5 mg DOC/L control in the PCA scores plot (Figure 1A) and there were no significant metabolite changes (Figure 2). This suggests that the presence of DOM did not substantially alter the metabolic profile of *D. magna* to TCEP exposure. Sorption coefficients to DOC (Log K_DOC_) were calculated for the three OPEs using an equation derived from experimental data of the sorption of neutral polar compounds to natural DOC sources (Table 1) [10]. TCEP has the lowest calculated Log K_DOC_ value (Log K_DOC_ = 1.81), which suggests that TCEP has the weakest sorption to DOC (Table 1). The calculated Log K_DOC_ values are predictions only and can differ from experimentally measured Log K_DOC_ values to various sources of DOM. For instance, experimentally determined Log K_DOC_ values for TCEP sorption to different sources of DOC ranged from 3.89–4.29 [8]. TCEP has an overall weaker sorption to DOM compared to highly hydrophobic OPEs [8], therefore DOM was not hypothesized to alter the bioavailability and subsequent metabolic response to TCEP exposure. Overall, the addition of DOM to TCEP exposure did not change the metabolic response of *D. magna* to TCEP exposure.

TBOEP, which has a higher Log K_OW_ than TCEP, is more likely to sorb with DOM which may reduce the free concentration of TBOEP in water [37] (Table 1). The predicted Log K_DOC_ also suggests that the free concentration of TBOEP should be reduced in the presence of DOM (Log K_DOC_ = 3.18), which may lessen the metabolic response of *D. magna* if TBOEP toxicity is related to the exposure concentration of TBOEP. The PCA scores plot shows that the TBOEP with 5 mg DOC/L treatment group did significantly (*p* < 0.05) separate from the 5 mg DOC/L control (Figure 1B). The separation of the TBOEP with 5 mg DOC/L exposure group from the 5 mg DOC/L control in the individual PCA scores plots (Supplementary Materials Figure S4C) is due to the metabolites valine, alanine and leucine that were separated on the corresponding PCA loadings plot (Supplementary Materials Figure S4D). TBOEP with 5 mg DOC/L exposure also resulted in significant (*p* < 0.05) increases in nine amino acids and a significant (*p* < 0.05) decrease in glucose (Figure 3), similarly to what is observed with TBOEP only exposure. Therefore, the toxic mode of action to TBOEP exposure remains the same with the addition of DOM where there is a decrease in immediate energy reserves in the form of glucose and an increase in amino acids which suggests the break-down of proteins for energy [16,33]. Therefore, this indicates that DOM was not able to mitigate the metabolic response to TBOEP exposure. Our results suggest that because TBOEP exposure has potent impacts on the *D. magna* metabolic profile, perhaps the freely dissolved concentration of TBOEP remaining in solution after sorption to DOM was able to elicit the same pattern of metabolic changes in *D. magna* as TBOEP exposure alone.

TPhP is the most hydrophobic OPE studied and based on its Log K_OW_ value and estimated Log K_DOC_ value, TPhP is hypothesized to have the highest sorption affinity for DOM (Table 1). Experimentally measured Log K_DOC_ values of TPhP to DOC from the Baltic Sea were also very high and averaged to 5.1 [38]. Therefore, it is anticipated that DOM will lower the bioavailability of TPhP and the extent of the metabolic response to TPhP exposure. The PCA scores plot showed that the TPhP with 5 mg DOC/L exposure group did significantly (*p* < 0.05) separate from the 5 mg DOC/L control along PC2 in the PCA scores plot (Figure 1C). Metabolite percent changes show there was a significant (*p* < 0.05) decrease in glucose and a significant (*p* < 0.05) increase in leucine from TPhP with 5 mg DOC/L exposure, which were not observed with TPhP only exposure (Figure 4). Therefore, the addition of DOM to TPhP exposure resulted in some metabolic changes and the overall metabolic profile from TPhP with 5 mg DOC/L exposure was significantly different compared to the DOM control. This contrasts the hypothesis of our study, which was that DOM would lessen metabolic disturbances from the exposure of hydrophobic organic contaminants, such as TPhP. The metabolic changes seen from TPhP with 5 mg DOC/L exposure could have occurred because there may not be complete sorption of TPhP to this DOM source, as this can be dependent on the type and concentration of DOM used [8]. For example, it was reported that Suwannee River humic acid did not impact the toxicity of TPhP to *D. magna* while Acros humic acid significantly decreased TPhP toxicity by over 50% [8]. This was likely because TPhP has a substantially higher sorption capacity for Acros humic acid (Log K_DOC_ is 3.83 ± 0.07) than for Suwannee River humic acid (Log K_DOC_ is 2.92 ± 0.46) [8]. In our study, DOM did not change the metabolic response to TCEP exposure and the inclusion of DOM did not change the mode of action to TBOEP exposure. However, DOM and TPhP together resulted in a unique metabolic profile which wasn’t consistent with either DOM or TPhP. Out of the three OPEs tested, TPhP has the highest predicted sorption to DOM (Table 1) [8]. Therefore, it should also be considered that DOM may have reduced the freely dissolved concentration of TPhP to an extent where a different metabolic response was seen with TPhP and DOM exposure compared to TPhP only exposure [8]. DOM may have enhanced the bioavailability of TPhP to *D. magna* [11]. A previous study conducted over 48 h with pyrene (60 µg/L) in the presence of 30 mg DOC/L found that the bioaccumulation of pyrene in *D. magna* increased by 42–92% compared to controls without DOM [11]. In addition, it was reported that DOM can enhance the water solubility of some hydrophobic organic contaminants likely due to a partitioning interaction with DOM, which may contribute to the increased uptake of organic contaminants in aquatic organisms [11,39]. Therefore, the freely dissolved concentration of TPhP may not be the only bioavailable form and DOM may have enhanced the bioavailability of TPhP to *D. magna* which contributed to the unique metabolic response observed.

In addition, although we did not observe a significant change in the *D. magna* metabolome with DOM alone, it may be that exposure to both DOM and TPhP resulted in unique biochemical perturbations. A previous study showed that exposure to Suwannee River natural organic matter with cypermethrin, cypermethrin alone or DOM alone changes the activities of the transformation enzyme systems of *D. magna* in different magnitudes and directions [31]. In addition, some DOM can act like a natural stressor to *Daphnia* and induce oxidative damage and reduce the amount of available energy [4]. Therefore, perhaps DOM and TPhP together may have acted as combined stressors that resulted in a distinct metabolic profile compared to TPhP only exposure.

## 3. Materials and Methods

### 3.1. Daphnia Culturing

*Daphnia magna* were originally obtained in 2013 from Ward Science Canada (St. Catherines, ON, Canada) and were subsequently cultured in our laboratory. The *D. magna* culture was maintained with a 16/8 h light/dark cycle at 20 °C and with dechlorinated tap water that was aerated for at least four days and has a hardness of approximately 120 mg/L as CaCO_3_. *Raphidocelis subcapitata* was grown in a Bristol medium and was used to feed *D. magna*. Feeding and a 50% water change were done every other day. Selenium and cobalamin (1 μg/L of each) were added to the culture water twice a week to ensure that the daphnids could meet the health criteria of Environment Canada [40].

### 3.2. Exposure to OPEs with DOM

TCEP (C_6_H_12_Cl_3_O_4_P, 97% purity), TBOEP (C_18_H_39_O_7_P, 94% purity) and TPhP (C_18_H_15_O_4_P, 99% purity) were purchased from Sigma-Aldrich (Mississauga, ON, Canada). Suwannee River natural organic matter was obtained as a freeze-dried, reference-grade sample from the International Humic Substances Society (St. Paul, MN, USA).

Dechlorinated tap water that was used for culturing *D. magna* was used for the control solutions where no external DOM was added. DOM solutions were made to a final concentration of 5 mg DOC/L using aged dechlorinated tap water and Suwannee River natural organic matter which has approximately 52% carbon [41]. Therefore, to yield nominal concentrations of 5 mg DOC/L, approximately 10 mg of Suwannee River natural organic matter was dissolved in one litre of aged dechlorinated tap water. The pH of DOM stock solutions was adjusted with dilute NaOH to 7.4 ± 0.3 to be consistent with the pH of the control solutions. Contaminant stock solutions of 150 mg/L TCEP, 150 mg/L TBOEP and 0.5 mg/L TPhP were also made with dechlorinated tap water. Correct volumes of contaminant stock solutions and DOM containing stock solutions were transferred to glass beakers to give final concentrations of either 23.5 mg/L TCEP, 14.7 mg/L TBOEP or 0.125 mg/L TPhP in the presence of 0 and 5 mg DOC/L. These sub-lethal exposure concentrations of TBOEP and TPhP are 10% of the 48-h LC_50_ values for *D. magna* (Table 1) [8,30]. The 48-h LC_50_ of TCEP to *D. magna* is not available in the literature and therefore 10% of the reported 24-h EC_50_ of TCEP to *D. magna* (Table 1) (EU, 2009) was chosen as the sub-lethal exposure concentration. Before the exposure of the daphnids, the beakers were sealed with parafilm and equilibrated on a magnetic stirrer at room temperature in the dark for 48 h. To consider the possible influence of DOM on *D. magna*, DOM control solutions without a contaminant at 5 mg DOC/L were prepared in parallel under the same conditions.

The stir bar was removed after 48 h of equilibration between the contaminant and DOM. *D. magna* adults that were 16 days old were then exposed to the chosen sub-lethal concentrations of either TCEP, TBOEP or TPhP in the absence or presence of DOM (0 and 5 mg DOC/L). Control exposures and DOM control exposures were done in parallel with the toxicant exposures. Each treatment group consisted of 10 replicate 500 mL beakers, and each beaker contained 10 daphnids at a density of 1 daphnid per 30 mL. *D. magna* were fed after 24 h from the start of the toxicity test with freeze-dried *R*. *subcapitata* at an amount of 0.1 mg of algae per daphnid to sustain a basal metabolic rate. Food was added midway through the toxicity test to avoid any physiological stress that food withdrawal may cause during organic contaminant exposure [42]. Therefore, all treatment groups received the same type and quantity of algae. Temperature and light conditions were the same as the culturing conditions. After 48 h from the start of the toxicity test the daphnids were removed from the exposure solution and rinsed in dechlorinated tap water. Next the daphnids were flash frozen with liquid nitrogen, lyophilized for 48 h and stored in a freezer at −25 °C until extraction. 

The concentrations of TCEP, TBOEP and TPhP used for the exposure experiments were measured at the start and after 48 h of the toxicity test using high performance liquid chromatography with tandem mass spectrometry (details are given in the Supplementary Materials). The measured concentrations of the OPEs were in agreement with the nominal concentrations except for TPhP which showed some minor losses (~15%) over the course of the experiment (Supplementary Materials Table S2).

### 3.3. Metabolite Extraction

The metabolite extraction procedure was based on a previously developed method for *D. magna* which uses 1.7 mm NMR tubes and a 1.7 mm microprobe to gather ^1^H NMR spectra which reduces the amount of dry mass needed from organisms [43]. Samples of *D. magna* dry mass were homogenized with a small metal spatula and weighed out to 1 mg subsamples with a microbalance (Sartorius ME36S, Geottingen, Germany) and placed into a 200 μL centrifuge tube. This process was done 10 times for each treatment group to obtain 10 replicates for NMR analysis. Next, 45 µL of a 0.2 M monobasic sodium phosphate buffer solution (NaH_2_PO_4_·2H_2_O, 99.3% purity, Fisher Scientific Company, Toronto, ON, Canada) was added to the dry mass in the 200 μl centrifuge tube. This buffer was prepared with D_2_O (99.9% purity, Cambridge Isotope Laboratories, Andover, MA, USA), 10 mg/L of 4,4-dimethyl-4-silapentane-1-sulfonic acid (DSS, 97% purity, Sigma Aldrich, St. Louis, MO, USA) as an internal calibrant and 0.1% *w*/*v* sodium azide (99.5% purity, Sigma-Aldrich) as a preservative [43]. NaOD (30% *w*/*w* in 99.5% D_2_O, Cambridge Isotope laboratories Inc. Andover, MA, USA) was used to adjust the pD of the D_2_O buffer solution to 7.4. Each sample was vortexed for 30 s followed by sonication for 15 min. The samples were then centrifuged (Eppendorf centrifuge 5804-R, Hamburg, Germany) for 20 min at 4 °C and 12,000 rpm (~15,000 g). The supernatant was pipetted into 1.7 mm Highthroughput^plus^ NMR tubes (Norell Inc., Morganton, NC, USA) for ^1^H NMR analysis.

### 3.4. ^1^H NMR Acquisition

A Bruker BioSpin Avance III 500 MHz NMR spectrometer equipped with a ^1^H-^15^N-^13^C TXI 1.7 mm microprobe fitted with an actively shielded Z gradient was used to acquire ^1^H NMR spectra. ^1^H NMR acquisition was completed with Presaturation Using Relaxation Gradients and Echoes (PURGE) water suppression [44], 256 scans, a recycle delay of 3 s, and 32 K time domain points. Spectra were apodized through multiplication with an exponential decay corresponding to 0.3 Hz line broadening in the transformed spectra, and a zero-filling factor of 2 [45,46]. All spectra were manually phased, baseline corrected and calibrated to the trimethylsilyl group of the DSS internal calibrant that was set to a chemical shift (δ) of 0.00 ppm.

### 3.5. Data and Statistical Analysis

The phased and baseline corrected ^1^H NMR spectra were analysed using AMIX software v. 3.9.14 (Bruker BioSpin, Rheinstetten, Germany). Analysis of the ^1^H NMR spectra was done in the proton chemical shift region of 0.5 to 10 ppm and the spectral region between 4.70 to 4.90 ppm was excluded due to the small residual H_2_O/HOD signals. The ^1^H NMR spectra were divided into 0.02 ppm buckets giving 475 buckets in total and the integration mode was set to the sum of intensities with the spectra scaled to total intensity [45,46,47]. The ^1^H NMR spectra were inputted into a PCA model. The resulting PCA scores belonging to each treatment group were imported into Microsoft Excel (v. 14. Microsoft Corporation, Redmond, WA, USA) and were averaged per group and replotted with their associated standard errors to make the PCA scores plots [45,46,47]. The statistical significance (*p* < 0.05) between the PCA score values of each treatment group were determined by a *t*-test. The statistically significant *(p* < 0.05) differences are of the OPE exposures compared to the control or of the OPE with DOM exposures compared to the DOM control, and are indicated by an asterisk (*) on the PCA scores plots. Individual PCA scores plots of the control and each OPE exposure group and their corresponding PCA loadings plots were also created with AMIX and are given in the Supplementary Materials.

Metabolites were identified using published spectra in the Madison Metabolomics Consortium Database [48] and metabolite resonance regions previously reported for *D. magna* [43]. A representative ^1^H NMR spectrum of the *D. magna* metabolome with all metabolites identified is given in Figure S6 in the Supplementary Materials. Metabolite percent changes of the OPE only exposure groups were calculated by subtracting the NMR intensity values of the OPE only exposure group from the corresponding bucket values of the control, then dividing this difference by the control bucket value [46,47,49]. Likewise, the metabolite percent changes of the OPE with DOM exposure groups were obtained by subtracting the NMR intensity values of the OPE with DOM exposure group from the bucket values of the DOM control, then dividing this difference by the DOM control bucket value. To evaluate if DOM exposure resulted in metabolic disturbances, the NMR intensity values of the DOM control group were subtracted from the bucket values of the control, and then this difference was divided by the control bucket value. The statistical significance of the individual metabolite percent changes was determined with a *t*-test (two-tailed, equal variances, *p* < 0.05) and the results of this statistical analysis is given in Table S3 in the Supplementary Materials

## 4. Conclusions

^1^H NMR-based metabolomics was used to evaluate the impact of DOM (5 mg DOC/L) on the metabolic response of *D. magna* exposed to three different OPEs. The *D. magna* metabolic response to the hydrophilic contaminant TCEP remained unaltered after the addition of DOM. The toxic mode of action of the hydrophobic contaminant TBOEP remained the same in the presence of DOM which suggests that the potency of TBOEP is high to *D. magna* that the addition of DOM was not able to alter the pattern of metabolic changes to TBOEP exposure. The addition of DOM to the exposure of the most hydrophobic contaminant TPhP resulted in a distinct metabolic response that was unlike DOM only or TPhP only exposure and may be due to combined metabolic stresses from DOM and TPhP exposure. These results suggest that Log K_OW_ may not always be a good predictor of how DOM will change sub-lethal contaminant toxicity due to a combination of DOM and contaminant impacts on the metabolome. To the best of our knowledge, this is the first study to use metabolomics to report how DOM changes the sub-lethal toxicity of OPEs. Future studies should investigate how DOM may alter the metabolic response of *D. magna* exposed to a mixture of OPEs at sub-lethal concentrations found in the environment. 

## Figures and Tables

**Table 1:** Physical-chemical properties of the selected organophosphate esters.

Compound and Chemical Structure	Aqueous Solubility (mg/L)	Log K_OW_	48-h *Daphnia magna* LC_50_ (mg/L)	Estimated Log K_DOC_
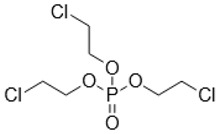 **Tris(2-chloroethyl) phosphate (TCEP)**	7000 ^a^	1.43 ^a^	24-h EC_50_ = 235 mg/L ^b^	1.81 ^c^
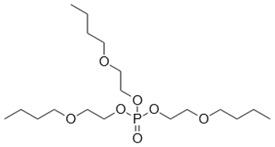 **Tris(2-butoxyethyl) phosphate (TBOEP)**	1100 ^a^	3.65 ^a^	147 mg/L ^d^	3.18 ^c^
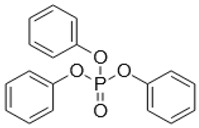 **Triphenyl phosphate (TPhP)**	1.90 ^a^	4.76 ^a^	1.25 mg/L ^e^	3.87 ^c^

^a^ From [28]. ^b^ From [29]. ^c^ From the equation Log K_DOC_ = (0.62 ± 0.03) × Log K_OW_ + (0.92 ± 0.19) in [10]. ^d^ From [30]. ^e^ From [8].

## Supplementary Materials

The following are available online at http://www.mdpi.com/2218-1989/8/2/34/s1, Table S1: The optimized multiple reaction monitoring transition related parameters for the three OPEs in positive ion mode using LC-ESI-MS/MS; Table S2: Nominal and measured OPE concentrations for the exposure period in test solutions. Measured concentrations are reported as averages with standard error from three replicated exposure experiments and duplicate analytical measurements; Table S3: Statistical results (*p* values) of the metabolite percent changes of *Daphnia magna* exposed to three OPEs in the absence and presence of DOM. Significant (*p* < 0.05) *p* values are marked by a * with *t*-test. The results of the DOM treatment are relative to the control without DOM, the OPE only treatments are relative to the control and the OPE with DOM treatments are relative to the 5 mg DOC/L control; Figure S1: Average principal component analysis (PCA) scores plot (A) of the ^1^H NMR spectra of *Daphnia magna* controls and 5 mg DOC/L controls (D5 Control). Scattered PCA scores plot (B) and the corresponding loadings plot (C) is shown. The chemical shift (ppm) in the ^1^H NMR spectra is represented by the numbers in the loadings plots. The metabolites responsible for the variation in the PCA scores plots are labelled in the loadings plots; Figure S2: The metabolite percent changes of *Daphnia magna* exposed to 5 mg DOC/L. The 5 mg DOC/L percent changes are relative to the control without DOM. Values are shown as mean ± standard error and * represents *p* < 0.05; Figure S3: Principal component analysis (PCA) scores from ^1^H NMR spectra of *Daphnia magna* controls and TCEP exposed treatments. (A) Control and TCEP or (C) 5 mg DOC/L control and TCEP with 5 mg DOC/L treatments and their corresponding loading plots (B) and (D). The chemical shift (ppm) in the ^1^H NMR spectra is represented by the numbers in the loadings plots. The metabolites responsible for the variation in the PCA scores plots are labelled in the loadings plots; Figure S4: Principal component analysis (PCA) scores from ^1^H NMR spectra of *Daphnia magna* controls and TBOEP exposed treatments. (A) Control and TBOEP or (C) 5 mg DOC/L control and TBOEP with 5 mg DOC/L treatments and their corresponding loading plots (B) and (D). The chemical shift (ppm) in the ^1^H NMR spectra is represented by the numbers in the loadings plots. The metabolites responsible for the variation in the PCA scores plots are labelled in the loadings plots; Figure S5: Principal component analysis (PCA) scores from ^1^H NMR spectra of *Daphnia magna* controls and TPhP exposed treatments. (A) Control and TPhP, (C) 5 mg DOC/L control and TPhP with 5 mg DOC/L treatments and their corresponding loading plots (B) and (D). The chemical shift (ppm) in the ^1^H NMR spectra is represented by the numbers in the loadings plots. The metabolites responsible for the variation in the PCA scores plots are labelled in the loadings plots; Figure S6: A ^1^H NMR spectrum of the *D. magna* metabolome from the control group with all identified metabolites labelled.
