# Supplementary material for: Investigation of Daphnia magna Sub-Lethal Exposure to Organophosphate Esters in the Presence of Dissolved Organic Matter Using 1H NMR-Based Metabolomics
Source: Metabolites. 2018 May 19;8(2):34. doi: 10.3390/metabo8020034 (PMC6027453; doi:10.3390/metabo8020034)
Supplement: Supplementary file 1 [file metabolites-08-00034-s001.pdf]

Supplementary Materials: Investigation of *Daphnia magna* Sub-Lethal Exposure to Organophosphate Esters in the Presence of Dissolved Organic Matter Using ^{1}H NMR-based Metabolomics

Vera Kovacevic, André J. Simpson, and Myrna J. Simpson

Concentration measurements of the organophosphate esters (OPEs)

The concentrations of the organophosphate esters (OPEs) used in the acute toxicity experiments were measured at the beginning and at the end of the 48-hour exposure. Aliquots from three replicate beakers were sampled and filtered through 0.2 μm Teflon syringe filters (Thermo Scientific, Rockwood, Tennessee, USA) prior to analysis. The OPE concentrations in the test solutions without dissolved organic matter (DOM) were quantified using liquid chromatography coupled to tandem mass spectrometry (LC-MS/MS). The same stock solutions of the OPEs were used for both contaminant-only and contaminant with DOM exposed treatment groups. When directly infused into a tandem mass spectrometer, natural DOM from the Suwannee River was previously shown to affect electrospray ionization and cause signal suppression in a suite of target analytes from endocrine disrupting compounds and pharmaceutical and personal care products [1].Therefore, because DOM may interfere with LC-MS/MS analysis, only samples from the contaminant without DOM exposed replicates were quantified.

The concentrations of the three OPEs in the test solutions without DOM were measured using an Agilent 1260 Series LC system coupled to an Agilent 6420 triple quadruple mass spectrometer [2]. The LC separation was performed using an Agilent ZORBAX Eclipse Plus C_{18} column (150 mm x 2.1 mm, 3.5-μm particle size), the column temperature was 45 °C and the injection volume was 5 μl. The flow rate was 0.25 mL/min for tris(2-chloroethyl) phosphate (TCEP) and tris(2-butoxyethyl) phosphate (TBOEP), while the flow rate was kept at 0.2 mL/min for triphenyl phosphate (TPhP) analysis. Eluent A was methanol/water (20/80) with 0.2% formic acid and eluent B was pure methanol with 0.2% formic acid. The OPE separation was done under gradient conditions as follows: 0 min (55% Eluent B), 0.5 min (70% Eluent B), 11 min (100% Eluent B) and 21 min (55% Eluent B). The mass spectrometer was operated in positive electrospray ionization mode with a capillary voltage of 3500 V. Quantification of all compounds was done by dynamic multiple reaction monitoring (MRM) mode and a cycle time of 500 ms was used with dwell times were automatically set using the cycle time. Quantification of the OPEs was performed using seven-point external calibration curves for each compound. The optimized MRM transition related parameters for the OPEs are listed in Table S1. The results of the measured OPE concentrations in the test solutions without DOM are listed in Table S2. By the end of the 48-hour exposure,

the TCEP and TBOEP concentrations were consistent with the amount added and nominal concentrations. TPhP concentrations did show small decreases over the 48-hour period but was still within 20% of the amount added and nominal concentration. TPhP hydrophobicity may have resulted in some minor sorption to algae or the daphnia themselves. There may have also been some sorption to beaker walls. However, the majority of the TPhP was recovered (84%) after 48-hours.

Table S1. The optimized multiple reaction monitoring transition related parameters for the three OPEs in positive ion mode using LC-ESI-MS/MS.

Compound	Parent ion (m/z)	Fragmentor voltage (V)	First product ion (m/z)	Collision energy of first product ion (V)	Second product ion (m/z)	Collision energy of second product ion (V)
TCEP	284.96	102	63.1	28	99	24
TBOEP	399.25	110	45.2	24	57.2	32
TPhP	327.08	148	77.1	48	152.1	40

Table S2. Nominal and measured OPE concentrations for the exposure period in test solutions. Measured concentrations are reported as averages with standard error from three replicated exposure experiments and duplicate analytical measurements.

Contaminant	Nominal concentrations	Measured concentrations at start of the exposure (0 hours)	Measured concentrations after 48 hours of exposure
TCEP	23.5 mg/L	25.0 mg/L ± 0.3 mg/L	26.7 mg/L ± 0.2 mg/L
TBOEP	14.7 mg/L	11.1 mg/L ± 0.8 mg/L	13.3 mg/L ± 0.5 mg/L
TPhP	0.125 mg/L	0.113 mg/L ± 0.003 mg/L	0.095 mg/L ± 0.002 mg/L

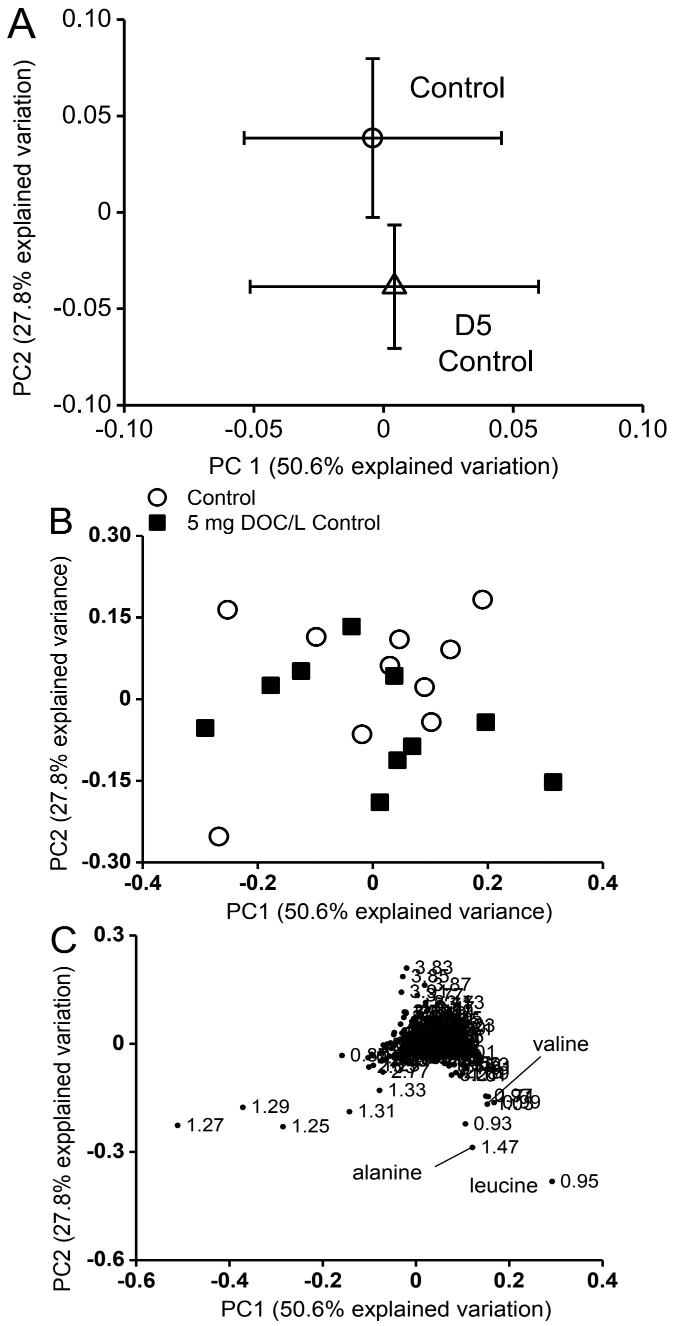

Figure S1. Average principal component analysis (PCA) scores plot (A) of the ^{1}H NMR spectra of *Daphnia magna* controls and 5 mg DOC/L controls (D5 Control). Scattered PCA scores plot (B) and the corresponding loadings plot (C) is shown. The chemical shift (ppm) in the ^{1}H NMR spectra is represented by the numbers in the loadings plots. The metabolites responsible for the variation in the PCA scores plots are labelled in the loadings plots.

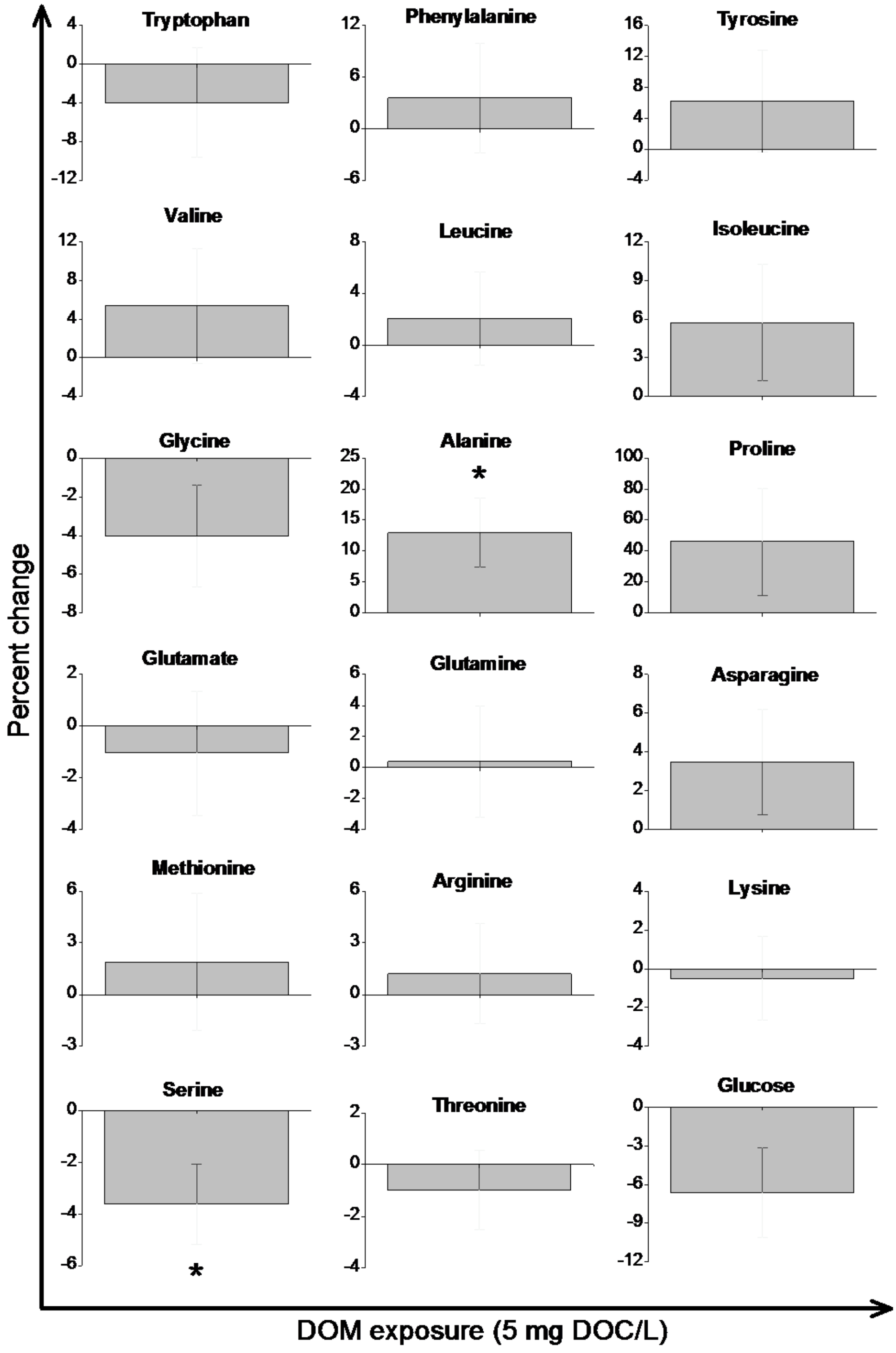

Figure S2. The metabolite percent changes of *Daphnia magna* exposed to 5 mg DOC/L. The 5 mg DOC/L percent changes are relative to the control without DOM. Values are shown as mean ± standard error and * represents $p < 0.05$.

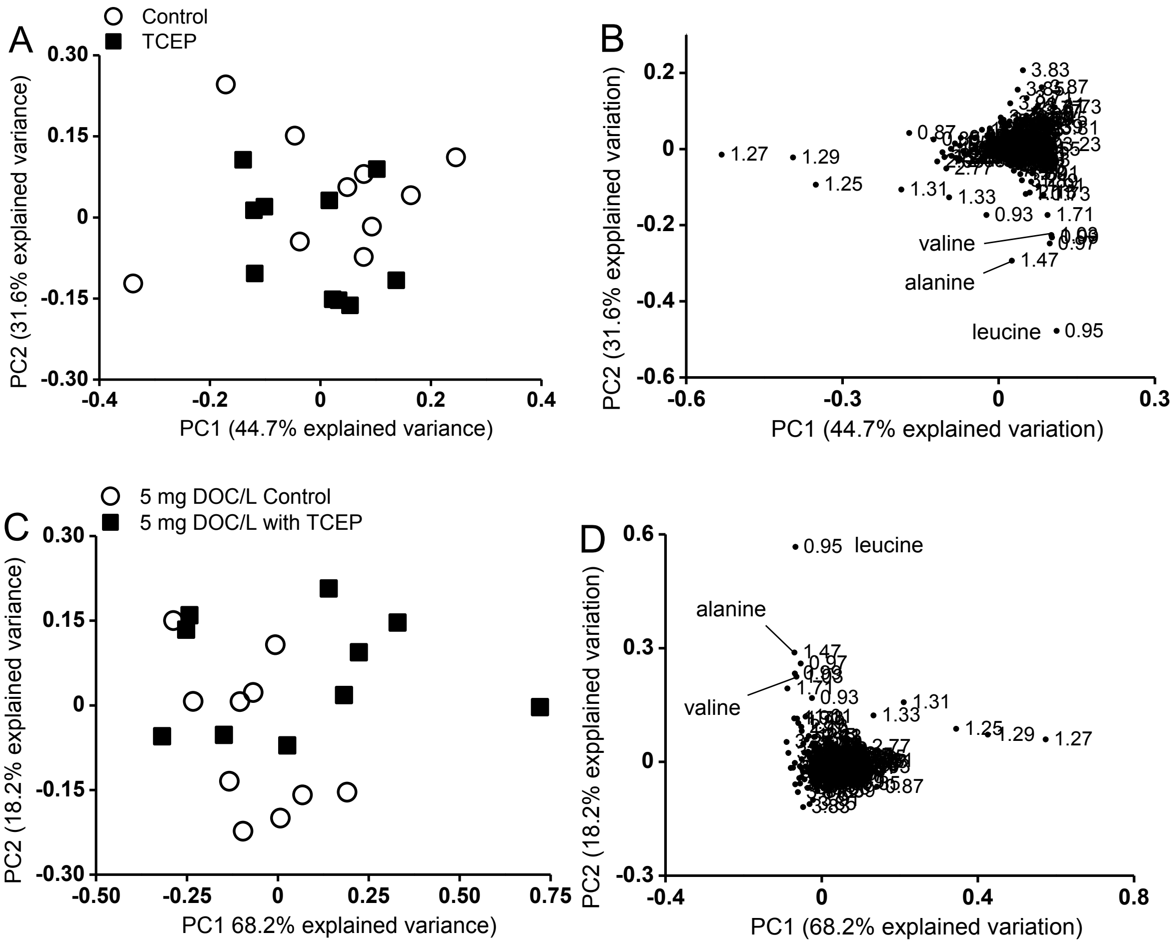

Figure S3. Principal component analysis (PCA) scores from ^{1}H NMR spectra of *Daphnia magna* controls and TCEP exposed treatments. (A) Control and TCEP or (C) 5 mg DOC/L control and TCEP with 5 mg DOC/L treatments and their corresponding loading plots (B) and (D). The chemical shift (ppm) in the ^{1}H NMR spectra is represented by the numbers in the loadings plots. The metabolites responsible for the variation in the PCA scores plots are labelled in the loadings plots.

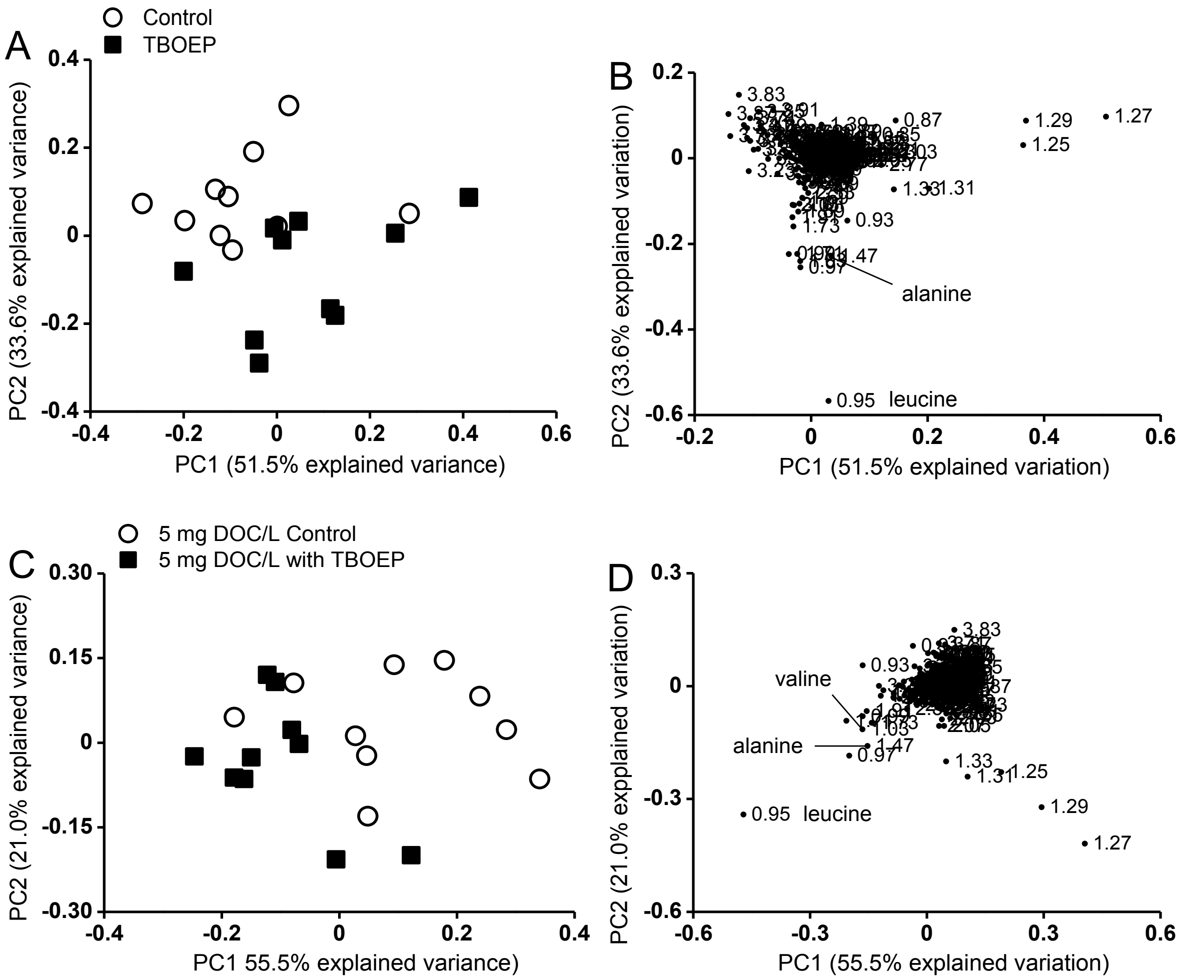

Figure S4. Principal component analysis (PCA) scores from ^{1}H NMR spectra of *Daphnia magna* controls and TBOEP exposed treatments. (A) Control and TBOEP or (C) 5 mg DOC/L control and TBOEP with 5 mg DOC/L treatments and their corresponding loading plots (B) and (D). The chemical shift (ppm) in the ^{1}H NMR spectra is represented by the numbers in the loadings plots. The metabolites responsible for the variation in the PCA scores plots are labelled in the loadings plots.

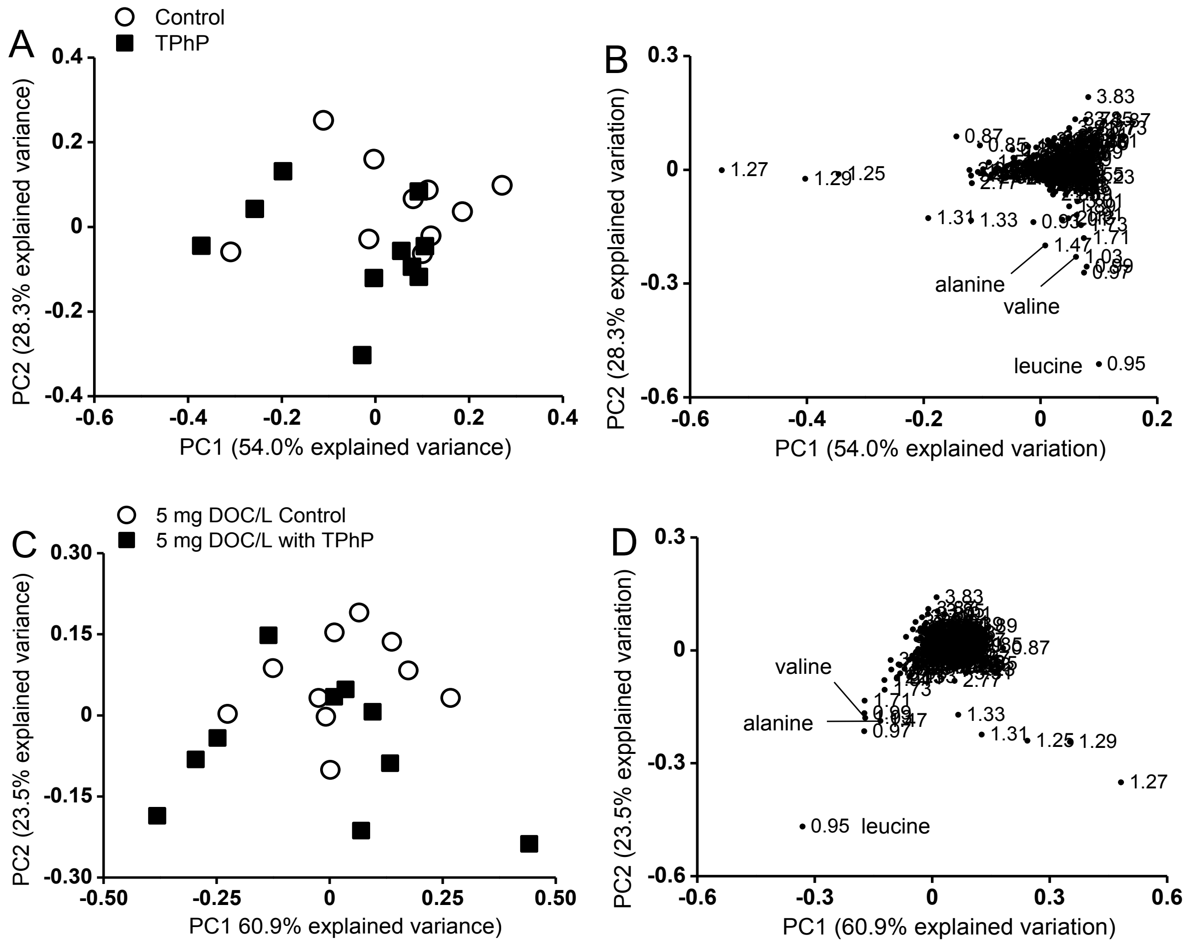

Figure S5. Principal component analysis (PCA) scores from ^{1}H NMR spectra of *Daphnia magna* controls and TPhP exposed treatments. (A) Control and TPhP, (C) 5 mg DOC/L control and TPhP with 5 mg DOC/L treatments and their corresponding loading plots (B) and (D). The chemical shift (ppm) in the ^{1}H NMR spectra is represented by the numbers in the loadings plots. The metabolites responsible for the variation in the PCA scores plots are labelled in the loadings plots.

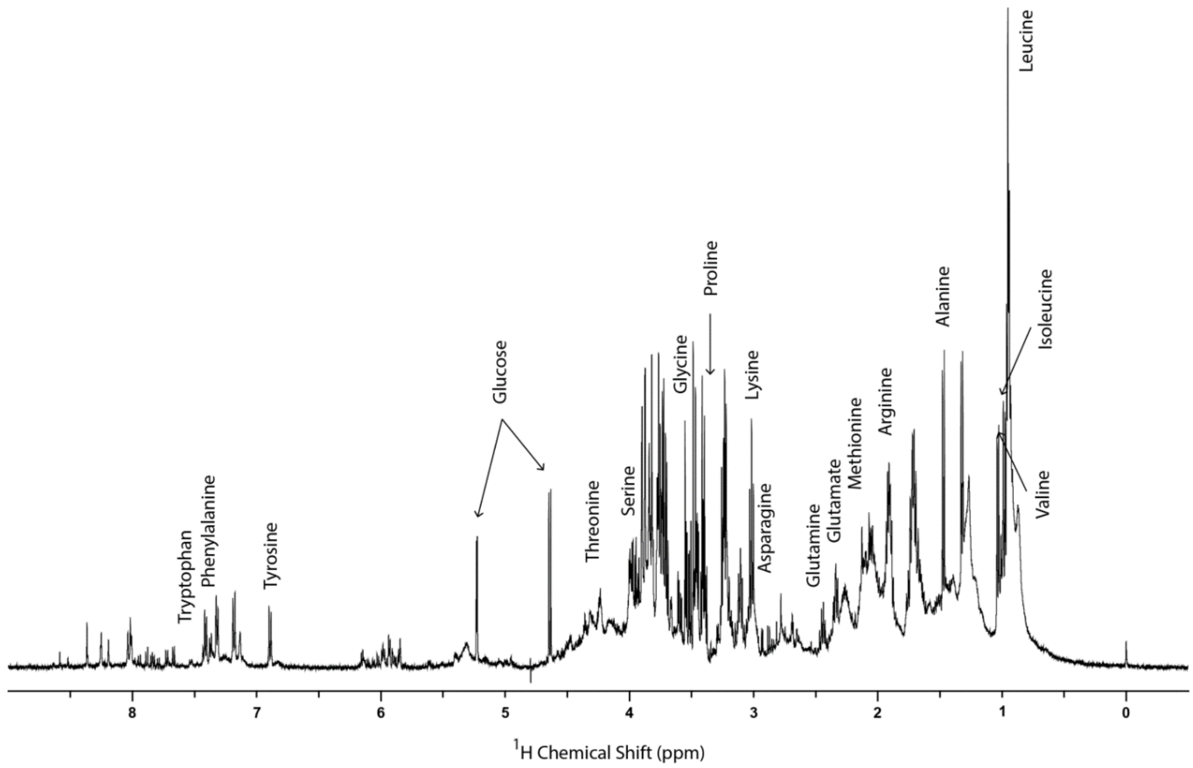

Figure S6. A ^{1}H NMR spectrum of the *D. magna* metabolome from the control group with all identified metabolites labeled.

Table S3. Statistical results (p values) of the metabolite percent changes of *Daphnia magna* exposed to three OPEs in the absence and presence of DOM. Significant ($p < 0.05$) p values are marked by a * with t-test. The results of the DOM treatment are relative to the control without DOM, the OPE only treatments are relative to the control and the OPE with DOM treatments are relative to the 5 mg DOC/L control.

Metabolite	DOM Control	TCEP only	TCEP with DOM	TBOEP only	TBOEP with DOM	TPhP only	TPhP with DOM
Tryptophan	0.4882	0.1784	0.5916	0.0887	0.0311*	0.4057	0.2106
Phenylalanine	0.5817	0.1096	0.2066	0.0141*	0.0042*	0.3243	0.0752
Tyrosine	0.3571	0.1364	0.7009	0.0052*	0.0065*	0.9703	0.3034
Valine	0.3741	0.1395	0.2848	0.0294*	0.0551	0.2955	0.1271
Leucine	0.5781	0.0198*	0.0562	0.0025*	0.0005*	0.1159	0.0396*
Isoleucine	0.2201	0.1197	0.5026	0.0713	0.1528	0.1233	0.1018
Glycine	0.1451	0.4097	0.1472	0.2292	0.1885	0.0344*	0.4897
Alanine	0.0307*	0.1778	0.3383	0.0348*	0.1893	0.4883	0.2467
Proline	0.1993	0.6499	0.4503	0.2323	0.4282	0.6111	0.3230
Glutamate	0.6672	0.6921	0.7820	0.1134	0.0343*	0.9115	0.3746
Glutamine	0.9162	0.7626	0.4862	0.1219	0.0015*	0.8410	0.1223
Asparagine	0.2153	0.8874	0.8677	0.1597	0.0291*	0.6809	0.2607
Methionine	0.6428	0.4984	0.8284	0.1776	0.1086	0.2292	0.1533
Arginine	0.6821	0.5345	0.5610	0.0210*	0.0012*	0.4928	0.1299
Lysine	0.8220	0.5372	0.5155	0.1075	0.0057*	0.7407	0.4655
Serine	0.0305*	0.2503	0.2217	0.1456	0.2127	0.0021*	0.1198
Threonine	0.5297	0.3974	0.1939	0.1816	0.7765	0.0161*	0.2832
Glucose	0.0715	0.2275	0.0727	0.0007*	0.0005*	0.1259	0.0427*

References

1. Wickramasekara, S.; Hernández-Ruiz, S.; Abrell, L.; Arnold, R.; Chorover, J. Natural dissolved organic matter affects electrospray ionization during analysis of emerging contaminants by mass spectrometry. *Anal. Chim. Acta* **2012**, *717*, 77-84.
2. Rodil, R.; Quintana, J. B.; Reemtsma, T. Liquid chromatography− tandem mass spectrometry determination of nonionic organophosphorus flame retardants and plasticizers in wastewater samples. *Anal. Chem.* **2005**, *77*, 3083-3089.
